# Supplementary material for: Patterns of Intron Gain and Loss in Fungi
Source: PLoS Biol. 2004 Nov 30;2(12):e422. doi: 10.1371/journal.pbio.0020422 (PMC532390; doi:10.1371/journal.pbio.0020422)
Supplement: Table S1 — Also available at http://genes.mit.edu/NielsenEtAl/. (4.3 MB ZIP). [file pbio.0020422.st001.zip › NielsenEtAl/html/69.html]

AN5856.1.NCU03312.1.MG09884.1.FG00371.1


```
 CLUSTAL W (1.82) Multiple Sequence Alignments - Introns Inserted


Sequence 1: NCU03312.1	822 aa
Sequence 2: MG09884.1	813 aa
Sequence 3: FG00371.1	836 aa
Sequence 4: AN5856.1	836 aa
Alignment Length: 898 aa
Number Identitical Residues: 285 aa
Alignment Score (without introns) 14449


MG09884.1 	--------------------------MEASSCHT--------EQQPGRRKRTHRGGKKKK
NCU03312.1	------------------MDLNTPPRHDASASNSGPSAGQPPQNQPSRRRKNHRAGKKKK
FG00371.1 	-----MSSNRYTDLANPSRSAAAPPASDPNQSQAPESSNNSNASRAARKRKGHRGGRKKR
AN5856.1  	MSRPLPTSLQNSPFPNKSTSGEVRSSGAPAEDDLPPMQTGQDLGTGSKKHKRKKNRNRKR
          	 : .  ::   :  .. : .    .   .   .             .:::: ::  .:*:

MG09884.1 	KSRRQSFATGPDDMAND---------------DLSTAGSNFYTNHAAHLSNTSLDSEALL
NCU03312.1	KNRRTSFGVTSEDMAQERMND----------KALAEARNGFYSQPGNNFSNTSIDSEALL
FG00371.1 	-TRRKSFAAMDDDDDHD--------------ETREAAGEGFFNLPRANLSNTSIDSEALL
AN5856.1  	RNRRPSFLAPEDSQPNAPPTIPESEALSAMAENQAKARGSTPFYSGGNMSNTSLESEALL
          	 .** ** .  :.  :   . ..:.: :: :.    *  .       ::****::*****

MG09884.1 	DHR~DQQ-SLRPRRPSIAVTSASPFPSNTQPYSSSTSRLRTVYHGGESNSADEDDHSANE
NCU03312.1	DHR2EQQPFLRPRRPSMLVSQSSHLTT-----PQVASRLKTVVASS-NNSEDE-----HE
FG00371.1 	DHR2DHQ-PLRPRR-TSTVPLPGGMSVSSPFSSSAGNRLRSIPAVS-AEEEHQDHTQWDE
AN5856.1  	DHR2NQP-MMRPRRESRLAQSFRPGSMSTSFRDSDSRPFHSRGKSTLENDSDQED--VND
          	*** ::   :**** :  .      . .:    .    :::       :. .:.    .:

MG09884.1 	GAPLLSQSMRLGNSEAALGYGSREMTARESTSRSRTSSGMSVDKKKLHLNPLSTSNNSYN
NCU03312.1	GAPLLSSSVFQ-RSEGFAGYGSNDTARRDSVA-SRLESSRDSSKRRRATSLGATGTEGYN
FG00371.1 	NAPLLGESIIS-------GYGAS--DARSNRTRSRRPSNKS-STTRLGTSSGARDNN-YD
AN5856.1  	RTPLMRPPSGR--IPKSPGYGTDSVASPFTFHQRRRSTRSDASRCS------PRETLSHD
          	 :**:  .          ***: .     .    *  :  . .         .  . .::

MG09884.1 	VNYPPSMPGSPRMR-------PVDPVGLINFGDDLLRDEISRSE---------LRSRAES
NCU03312.1	VNYPPSVPGTPPMG-------ATDRDLSMSFGDIMMRDDVNSMP---------NSPGRRT
FG00371.1 	VNAPPSMPGSPTFG-------TADR-IELSLGDVMIRDGLGGRD---------DSPHRLM
AN5856.1  	INNPPSMPSTPKLGPDMGYNDAVMTGAEFDFGLGRSSDDRYDSSRPHDMVIDVEGTGRRS
          	:* ***:*.:* : .. . ....     :.:*     *       . .   .   .    

MG09884.1 	--IGDEERPSGRMSSPMERRHTISVPPEEDVCFPQDGMSEMGDDMAHNDSEDVHMGLRSK
NCU03312.1	--LNEESDLVG----SLERRHTIALQAEEDVCFPQDGLSEIADEDLRTRDP---TAYRMK
FG00371.1 	GEMDCLSDMDNMRDGRPDIGRRMATEAEQDVCFPVPGMSEIGDDDAQSQAH---EHRQYR
AN5856.1  	RSTPGSAAVSPHRTPQETLIRRRTVPAEEDVCFPQEDASEVAEECAPALSR-----GNGR
          	 .                  :  :  .*:*****  . **:.::             . :

MG09884.1 	GRRRKRRWPDLSVLEDWSKLEKEAN-EERRVKRITEPQLINGRLRPVNKT-WFKAEDDAP
NCU03312.1	-RRRRGKWPDLAILEEWSRYEKEGRSEERHAKKITEPQLINGRLRPVHRG-WYRAQEDAP
FG00371.1 	TRRRRGQWPDLSVLEEWSSMEKEDRTEERRVKRITEPQLINGRLRPIRKGQFYEAIEESP
AN5856.1  	RRRRTKEWPDLSILEDWSREEKEERIGVFRAKKISEPMLVEGRLRPQYRA-WRREEDEVP
          	 ***  .****::**:**  *** .    :.*:*:** *::*****  :  : .  :: *

MG09884.1 	YRFTYFNEEFQSTVHSQTISGLVQAGGTFRELFVPEPVIIEDSSSESDNDDMTSTIRQPT
NCU03312.1	YRFTYFNEEFQSTIHSQTISELVPPGGSFRQLFIPDPPLLSDSSDDEDDEEDQ---RHVG
FG00371.1 	YRFTYFNEEFQSTVHSQTISELVQPGGGFRELFVPDPRVLSDDESDDEDVEPILPTKLHN
AN5856.1  	YRFTYFNEEFQSTIHAQTIPELVQPGGSFRELFIPDPPELEDS-SDSEASESEEHRNSGH
          	*************:*:***. ** .** **:**:*:*  :.*. .:.:  :     .   

MG09884.1 	PGESRAPSRQTSVPPSIHASEHSSRPGYLNRTFSPPTGRDSVMTSAPP-ISGLETPVPPK
NCU03312.1	LDEVLGGQNGDPRIPSRQPSLTTTTQGYFDARRDRGSNAGTTVTDSKR-TSGEHTPTDTK
FG00371.1 	LS-----TEGNSKVHTRQPSLSTVHDNYEQREGTISPLKDPLAHVVSRSESGNATPTGRK
AN5856.1  	PTPSTRTANQSPAARAATPSNNHNHDTHQQPEG-RSQLRASIISEAVS-----EGRVSGD
          	        .  .   :  .*       : :          .               .  .

MG09884.1 	SPNSAKKSTTPPEG----KPVRFGERPVWWLDVLNPSEAEMRVISKTFGIHPLTAEDIIM
NCU03312.1	SPERSKSPPTEKTS----KPPKYGDRPVWWLDIYSPTEQEMKVISKAFGIHPLTAEDIMM
FG00371.1 	SADETAHVSQNTEGGSNEKMIRYGERPVWWLDVLCPTEAEMKVISKAFGIHPLTAEDIML
AN5856.1  	LPDQTQTAP---------KPKRYGPRPTFWLDVLCPTDAEMRVISKAFGIHALTAEDIMM
          	 .: :   .         *  ::* **.:***:  *:: **:****:****.******::

MG09884.1 	QEAREKVELFRHYYFVNYRTFDQDEESEDFLEPVNMYIVVFREGILTFHFSITPHPANVR
NCU03312.1	QEAREKVELFRHYYFVNYRSFDQDSASENFLEPVNMYVVVFREGVLSFHFSQTPHPANVR
FG00371.1 	QEAREKVELFRHYYFVNYRSFDQDEDTNNFLEPVDMYVVVFREGVISFHFSRTPHPANVR
AN5856.1  	QEAREKVELFRNYYFLNYRTFEQDPNSENYLEPVNMYVVVFREGILSFHFSQTPHPANVR
          	***********:***:***:*:**  ::::****:**:******:::**** ********

MG09884.1 	RRVRQLRDYMMLSADWISYAIIDDITDVFVPLITRIEEEVDDIDDEVLGLQEVSTAEAVK
NCU03312.1	RRIRQLRDYLILSSDWISYAIIDDITDVFGPLIHNIEDEVDDIDEAILLLHSEDDPNFPT
FG00371.1 	RRIRQLKDYLILSSDWISYAIIDDITDVFAPLIEKIETEVDDIDDAILRLHSSADKRLNE
AN5856.1  	RRIRQLMDYLILSSDWISYALIDDITDVFGPLIQSIEDEVDDIDEMIMHMHSDQSPASSS
          	**:*** **::**:******:******** ***  ** ******: :: ::.        

MG09884.1 	DTTSFKSKD---------NAMLRRIGDCRKKVMGLYRLLGNKADVIKGFAKRCNEHWEVA
NCU03312.1	DNLKEKSEAGDATSAESGRDMLRRVGDCRKKVMSLYRLLGNKADVIKSFAKRCNEHWEVA
FG00371.1 	ESSKNDDAMAAS---DSNIDMLQQVGECRKKVMKLYRLLGNKADVIKGFAKRCNERWEVA
AN5856.1  	PKEEQRDVVPGP------GEMLRRVGVCRKKVMGLYRLLSNKADVVKGFAKRCNEHWEVA
          	 . .  .    .        **:::* ****** *****.*****:*.*******:****

MG09884.1 	PRSDIGLYLGDIQDHIVTMTANLSHYEK~ILARSHANYLAQISLRMNERQEETADILGKL
NCU03312.1	PRSEIGLYLGDIQDHIVTMTSNLSHYEK2ILARSHANYLAQINIRMNERQEQTADVLGRL
FG00371.1 	PRSEIGLYLGDIQDHIVTMTANLGHYEK2ILARSHGNYLAQINIRMNERQEQTADVLGKL
AN5856.1  	PKSEIGLYLGDIQDHIMTMTSSLTYYET2LLSRAHSNYLAQINIHMNERQEQTADVLGKL
          	*:*:************:***:.* :**. :*:*:*.******.::******:***:**:*

MG09884.1 	TILGTIVLPMNLVTGLWGMNVWVPGQAAEGDLTWFFAI~TVSLLSAGLLCYWLVKKLYGL
NCU03312.1	TVLGTIVLPMNIVTGLWGMNVWVPGQGNEGDLKWFTAI~TAGLLLFGFGCYLIAKRVYKI
FG00371.1 	TVLGTIVLPMNIITGLWGMNVWVPGQEYEGDLQWFFWI~TAGLLFFGFACFMIAKRVYNI
AN5856.1  	TVLGTIVLPLNIIC-----------------------V1TGGLIIFAFASFLIAKRVYNI
          	*:*******:*::                        : * .*:  .: .: :.*::* :

MG09884.1 	-
NCU03312.1	V
FG00371.1 	V
AN5856.1  	V
          	
```
